# Habit and Identity: Behavioral, Cognitive, Affective, and Motivational Facets of an Integrated Self

**DOI:** 10.3389/fpsyg.2019.01504

**Published:** 2019-07-10

**Authors:** Bas Verplanken, Jie Sui

**Affiliations:** Department of Psychology, University of Bath, Bath, United Kingdom

**Keywords:** habit, identity, integrated self, true self, self-esteem, self-regulatory focus, value affirmation

## Abstract

Two studies investigated associations between habits and identity, in particular what people consider as their “true self.” Habit-identity associations were assessed by within-participant correlations between self-reported habit and associated true self ratings of 80 behaviors. The behaviors were instantiations of 10 basic values. In Study 1, significant correlations were observed between individual differences in the strength of habit-identity associations, measures of cognitive self-integration (prioritizing self-relevant information), self-esteem, and an orientation toward an ideal self. Study 2 further tested the assumption that habits are associated with identity if these relate to important goals or values. An experimental manipulation of value affirmation demonstrated that, compared to a control condition, habit-identity associations were stronger if participants explicitly generated the habit and true self ratings while indicating which values the behaviors would serve. Taken together, the results suggest that habits may serve to define who we are, in particular when these are considered in the context of self-related goals or central values. When habits relate to feelings of identity this comes with stronger cognitive self-integration, higher self-esteem, and a striving toward an ideal self. Linking habits to identity may sustain newly formed behaviors and may thus lead to more effective behavior change interventions.

## Introduction

What determines our identity? A potential source of identity, which has received little attention in the literature on the self and the self-concept, is the array of our habits. A large portion of everyday behavior is habitual, that is, being performed frequently, often automatically, and in stable contexts (e.g., Verplanken and Aarts, 1999; Wood et al., 2002; Gardner, 2015; Verplanken, 2018). Habits vary in a number of ways. One is complexity; some habits involve simple acts, such as nail biting or checking the time, while others are part of more complex behaviors or routines, such as donating to charity or exercising. Habits also vary in terms of involvement of other people. For instance, taking the car to work is a solitary activity, whereas calling your parents maintains a relationship. And habits vary in the extent to which they are important to us. We may not even be aware of the many *un*important habits, such as where you sit at the table or the way you tie your shoes. Other habits are more important, such as those which express an important value. An unanswered question is whether or when habits contribute to what we consider as our identity, and if this is the case, how these sources of identity are embedded in other self-related constructs and processes, such as beliefs about ourselves, self-esteem, and self-regulation.

Personal or self-identities can be considered as mental representation individuals hold about who they are, which include autobiographical memories, self-attributions, beliefs, motivations, recurrent thoughts, emotions, and self-perceptions. These narratives are constantly constructed and revised (e.g., Vignoles, 2011). Habits may become part of self-identities through various psychological processes. One such process may be the end result of enacted motivations, such as suggested in socio-cognitive models (e.g., Fishbein and Ajzen, 1975; Deci and Ryan, 1991; Rise et al., 2010). A strong motivation, anchored in self-identity, may instigate repeated action, which may then become a habit. Such habits may function as vehicles of self-control in accomplishing a goal: habits relieve an individual from having to deliberate and decide on actions and may thus promote the accomplishment of a goal (e.g., Galla and Duckworth, 2015). Another path to a habit-identity relation is through self-perception (e.g., Bem, 1972). Through the perception of our own frequently performed behaviors, we may infer that these are important to us and may thus be part of who we are (e.g., Neal et al., 2012; Wood and Rünger, 2016).

### Empirical Evidence for a Habit-Identity Relation

What is the evidence for a habit-identity relationship? Some habits directly signify a particular identity. For instance, while the culture around smoking is rapidly changing, in some population segments this habit still stands for masculinity or being “cool” (e.g., Ng et al., 2007). Self-identity has been studied as a potential addition to the theory of planned behavior. This theory poses an intention to act as the primary determinant of behavior, which in turn is determined by an attitude, normative pressure, and perceived control of the behavior (e.g., Ajzen, 1991). In a meta-analysis on the role of self-identity in the theory of planned behavior, Rise et al. (2010) established that self-identity correlated 0.33 with past behavior, which has often been considered as a proxy for habit. A number of primary studies provided evidence for a habit-identity association. Charng et al. (1988) reported a 0.22 correlation between blood donation habit and a measure of identity as blood donor. Gardner et al. (2012) found a strong correlation between measures of binge drinking habit and binge drinking identity among university students (*r* = 0.69). Gardner and Lally (2013) found a strong correlation between habit and intrinsic motivation for physical activity (*r* = 0.64). Gatersleben et al. (2014, Study 2) found that measures of environmental and frugal identities mediated between environmental values and pro-environmental behaviors (*β*s = 0.35 and 0.28, respectively). Lindgren et al. (2015) found a significant correlation between an implicit measure of drinking identity and drinking habit (*r* = 0.36). Verplanken and Roy (2016) found that an index of pro-environmental habits correlated significantly with biospheric values (*r* = 0.31), personal norms (*r* = 0.45), and personal involvement (*r* = 0.30), i.e., constructs that are closely related to self-identity. McCarthy et al. (2017) reported a strong correlation between a measure of health-conscious identity and an assessment of healthy eating habit (*r* = 0.69). Albini et al. (2018) found a significant correlation between personal importance and habit of consuming vegetables (*r* = 0.49). As the relationships mentioned above are correlational, the causal flow in the habit-identity relation is unknown and may well be bi-directional: a particular identity may instigate behavior and thus maintain a habit, while the self-perception of a habit may feed into self-identity (cf., Wood and Rünger, 2016).

Two perspectives outside the social psychological domain may be taken to support a link between habit and identity. The first comes from the area of moral development of the self as a core of personal identity. Developing a self-concept and self-identity comes with the development of a moral identity (e.g., Blasi, 1994). From an early age, we learn to “do the right thing” in a variety of situations. By repeating such moral actions, these may turn into moral habits and feed into a moral identity. Such habits may become what can be designated as “character,” “second nature dispositions,” or indeed, a moral identity (e.g., Aquino and Reed, 2002; Hulsey and Hampson, 2014; Ward and King, 2018). Second, an interesting view on the relationship of habit and identity from a philosophical perspective was put forward by Wagner and Northoff (2014). These authors discussed the difference between “personhood” and “personal identity.” Personhood refers to features that define a person at one specific point in time. However, as such features are fluid and impermanent, and in order to persist as the *same* person, that is, to have a personal identity, features need to remain stable. Wagner and Northoff (2014) thus considered habit as an explanatory construct, which links these different temporal dimensions to form a personal identity.

The empirical basis of a relationship between a habit and a self-identity is not unequivocal. For instance, Murtagh et al. (2012) reported nonsignificant correlations between a measure of identity and measures of past travel mode behaviors (*r*s varying between 0.02 and 0.07). Also, while in the Albini et al. (2018) study cited above personal importance and habit of consuming vegetables correlated significantly, no such correlation was present for consuming fruit (*r* = 0.06). There was neither evidence of a habit-identity relation in a comprehensive study into the nature of students’ everyday habits conducted by Wood et al. (2002), in which participants were asked to write hourly reports on their ongoing behaviors and experiences. If anything, in this study habits were associated with negative self-evaluations and the relative *un*importance of these behaviors for attaining personal goals.

Taken together, the studies and perspectives discussed above lead to two conclusions. The first is that there exists significant, and sometimes substantial, associations between measures of habit and measures of self-identity, and there are some arguments beyond social psychology for such a relationship. Second, such correlations are not being found across the board; there is a large variation between studies in the size of habit-identity correlations. This suggests that certain habits, but not all, relate to self-identity. We contend that prime candidates for such a role are habits that are related to important goals or values. Goals and values may be integrated in one’s self-concept and are thus likely to be repeatedly enacted (e.g., Deci and Ryan, 1991; Sheldon and Elliot, 1999; Aarts and Dijksterhuis, 2000; Verplanken and Holland, 2002; Bardi and Schwartz, 2003; Hitlin, 2003; Gatersleben et al., 2014; Burkley et al., 2015). In addition, we anticipate that people differ in the strength of habit-identity associations. First, different people have different habits and may thus associate different habits with their self-identity, which may lead to variation between studies. Second, people may differ in the extent to which they identify habits as being relevant for one’s identity in the first place.

### The Integrated Self

An emerging theme in the literature on the self is the realization that some parts of the self are more essential than others, which has been referred to as real self (e.g., Rogers, 1961), authentic self (e.g., Koole and Kuhl, 2003; Johnson et al., 2004), or true self (Newman et al., 2014; Strohminger et al., 2017). At the heart of this concept lies the notion of an *integrated self*, that is, a high degree of connectedness within and between cognitive, affective, motivational, and behavioral systems. Kuhl et al. (2015) presented a neurobiological model, which explains the various functional characteristics of the integrated self, such as emotional and somatosensory connectedness, attention to self-relevant information, and self-positivity. The integrated self is holistic and incorporates a vast amount of autobiographical memory. It functions by means of high-level parallel-distributed processing, operating largely at implicit levels, and is thus able to integrate a large amount of self-related processes – cognitive, emotional, motivational, and volitional – simultaneously (Kuhl et al., 2015). We contend that self-perception of behaviors *per se* is not what connects them to the self but that behaviors become part of the integrated self if two conditions are fulfilled. One is that the behavior has become habitual, that is, being repeatedly and automatically executed and has thus become ingrained in the person’s autobiographical memory. The second is that a behavior is related to an important goal or value. This is not the case for all habits and for all individuals.

Sui and Humphreys (2015) summarized a body of work that sheds light on properties of an integrated self in more detail at the neuro-cognitive level. As an indicator of the degree to which a person possesses an integrated self, these researchers used perceptual matching tasks, which assess differences in reaction time and accuracy between matching self-related versus other-related stimuli (Sui et al., 2012). Larger differences indicate stronger self-prioritization effects (i.e., a stronger “self-bias”). Sui and colleagues demonstrated that self-referencing can have wide-ranging integrative effects with respect to perception, attention, memory, and decision making (e.g., Sui et al., 2012), which is thus interpreted as cognitive self-integration. This evidence suggests that self-referencing is not simply a narrative reflecting ongoing self-related processes. Rather, self-referencing actively modulates cognitive processes and acts as a “glue,” which binds different forms of information, for instance, between stimuli in perception and memory, or integrates different stages of information processing, such as in decision making. Sui et al. (2012) argued that self-referencing leads to robust self-prioritization effects in perception and cognition. Sui and Gu (2017) further put forward a neural framework of an integrated self where they argued that cognitive and affective aspects of the self-interact to influence behavior through the three neural networks – the ventral network including the ventral medial prefrontal cortex (vmPFC), the cognitive control, and the salience networks. Researchers have reported that inducing emotional valence can alter self-prioritization in face recognition. For example, when participants are asked to evaluate negative personality traits, there is a reduced advantage for processing self vs. others’ faces (Ma and Han, 2010). Consistent with this, the self-prioritization effect in the perceptual matching task was disrupted in individuals with low mood (Sui et al., 2016), due to the breakdown of the integrated self (in this case, the intrinsic association between self and positive emotion) in depressed individuals. In short, the strength of self-prioritization (“self-bias”) observed in these perceptual matching tasks can be considered as a proxy for cognitive self-integration.

At the experiential level, a number of authors describe the “true self,” which arguably is a subjective experience of an integrated self (e.g., Newman et al., 2014; Strohminger and Nichols, 2014; De Freitas et al., 2018). The true self is what a person considers as one’s authentic core and is experienced as inherently moral and good. Although the true self is in essence a belief a person holds about oneself and may thus be false or distorted, it has consequences for a person’s cognitive and social functioning. For example, it has been reported that unfavorable self-related events are more likely to be forgotten (Hu et al., 2015). People also tend to attribute positive outcomes to themselves relative to other people while linking negative outcomes to others, thus demonstrating biased causal attributions in social evaluation (Greenwald, 1980), or to influence the environment (e.g., Newman et al., 2014). Finally, moral values that make up someone’s true self may serve as benchmarks to judge others’ moral value status (e.g., Newman et al., 2014). Thus, the true self has the potential to evoke feelings of self-worth and a sense of meaning in life (e.g., Schlegel et al., 2009) and to protect the self from negative perspectives (Sedikides and Green, 2009). Particular habits, then, may be seen as instantiations of the accomplishment of goals or values associated with the true self and may thus become incorporated in one’s self-identity.

### The Present Studies

The present studies aimed at investigating the relationships between the degree to which individuals associate habits with their true self and how this relates to cognitive, affective, and motivational aspects of the self. Variation in habit-identity associations was assessed by presenting participants with 80 behaviors, and asking two ratings for each of those behaviors, i.e., self-reported habit and how much the activity reflects their true self. For each participant, a correlation was calculated between these two ratings across the 80 behaviors, which thus served as a measure of habit-identity associations. In Study 1, this association measure was correlated with the measures of cognitive self-integration obtained by the perceptual matching paradigm as developed by Sui et al. (2012). In addition, the study contained assessments of self-esteem as an affective component of the self and chronic self-regulatory focus style (i.e., “promotion” and “prevention”; Higgins, 1998) as a motivational aspect of the self. A promotion style is an orientation toward hopes, aspirations, and your ideal self. A prevention style is an orientation toward safety and responsibilities and fulfills what you think ought to be done. Positive correlations were expected between habit-identity associations, cognitive self-integration, self-esteem, and a promotion-style self-regulatory focus. Study 2 focused in more detail on the habit-identity association measure. This study aimed at demonstrating that habit-identity associations are stronger if these are being generated in the context of goals and values compared to a more concrete context.

## Study 1

### Method

#### Participants and Procedure

The study was conducted in a laboratory at the authors’ university. A power analysis was conducted prior to this study. In a previous study among 67 participants, admittedly older than in the present study, a mid-range correlation of 0.36 (*p* < 0.003) was found between cognitive self-integration in the perceptual matching task used in the present study and a self-report measure of personal distance (Sui and Humphreys, 2017). Together with setting an *α* of 0.05, two-sided testing, accepting a power of 0.80, and aiming at detecting medium effect size correlations (*r* ≈ 0.30), a sample size of approximately 85 was required. A total of 90 participants were recruited from the university’s student population. There were 29 males and 61 females. Their mean age was 21 years (SD = 2.67). All participants had normal or corrected-to-normal vision. Informed consent was obtained from all participants according to procedures approved by the authors’ departmental ethics committee (IRB).

Participants worked individually and visually separated. They first carried out the perceptual matching task, which assessed cognitive self-integration. This was followed by a questionnaire, which contained the habit and identity ratings and assessments of self-esteem and self-regulatory focus. A session took 30–40 min. Participants were paid £5.00 for their contribution.

#### Measures

##### Cognitive Self-Integration

Cognitive self-integration was measured by assessing the strength of self-prioritization (“self-bias”) in a perceptual matching task (Sui et al., 2012). Participants were first asked to name one of their best friends. They then selected a gender-matched stranger from a common name list not corresponding to anyone they knew. The named friend and stranger were then used in the perceptual matching task, where they were instructed to associate three geometric shapes (triangle, circle, square) with labels indicating the self (“You”), the named best friend (“Friend”), and the named stranger (“Stranger”), respectively. The assignment of the particular shapes to the three labels was counterbalanced across individuals. The self-prioritization scores were calculated using the performance scores of “You” and “Stranger.” The reason “Friend” was included in the task was to make it sufficiently challenging so as to avoid ceiling effects.

After the association instruction, participants conducted the shape/label matching task. Participants were asked to judge whether or not simultaneously presented shape/label pairs (e.g., a circle/“You”) matched according to the associations they had been instructed to make. Each trial started with a central fixation cross for 500 ms, followed by a shape/label pair at the center of the screen for 100 ms. A shape (triangle, circle, or square) with 3.5 × 3.5° of visual angle appeared above a white central fixation cross with 0.8 × 0.8° of visual angle. One of three labels (“You,” “Friend,” or “Stranger”) covering 1.76/2.52° × 1.76° of visual angle was displayed below the fixation cross. All stimuli in white were displayed on a gray background. E-prime software version 2.0 was used to present the stimuli and to record responses. The experiment was run on a PC with a 22-in monitor (1,920 × 1,080 pixels) at 60 Hz.

Half of the shape/label pairs conformed to the association instruction and should thus be responded to as “match” trials; on the remaining trials, the shapes and labels were re-paired to form “mismatch” trials. For mismatch trials, a shape was paired with one of the other labels (e.g., a circle/“Stranger,” in our example). The next frame was a 1,000 ms blank field. Participants were encouraged to make a “match” or “mismatch” response as quickly and accurately as possible within this 1,000 ms interval by pressing one of two keys on the keyboard with the index or middle finger of the right hand. The order of response keys was counterbalanced across participants. A feedback message (“correct,” “incorrect,” or “too slow”) was then given in the center of the screen for 500 ms. Participants were informed of their overall accuracy at the end of each block. There were three blocks of 60 trials following 12 practice trials. Thus, there were 30 match and 30 mismatch trials in each block.

Self-bias scores were calculated for reaction times (RT) and accuracy, respectively, for correct responses on match shape-label trials. Only correct responses longer than 200 ms were included. All participants had accuracy scores >0.55 (i.e., 5% or more above chance level). Self-bias on RT was inferred from the difference in RT for the self against the stranger condition, divided by the sum of the two conditions and multiplied by 100 {i.e., 100 × [(stranger − self)/(self + stranger)]}. Self-bias on accuracy was indexed by the difference in performance for the self against the stranger condition divided by the sum of the two conditions [i.e., (self − stranger)/(self + stranger)]. Larger scores of both measures indicated a stronger self-bias and thus were taken as stronger cognitive self-integration.

##### Habit-Identity Associations

Participants were presented with 80 behaviors, which were chosen to cover 10 value-related motivation areas (cf., Schwartz, 1992; Bardi and Schwartz, 2003): hedonism (e.g., “Enjoy a movie”), stimulation (e.g., “Do something exciting”), self-direction (e.g., “Find something out by yourself”), universalism (e.g., “Buy ecological products”), benevolence (e.g., “Donate to charity”), conformity (e.g., “Wear what’s in fashion”), tradition (e.g., “Attend family occasions”), security (e.g., “Make sure your door is locked”), power (e.g., “Make your voice be heard”), and achievement (e.g., “Study during the weekend”). Participants were asked to provide two ratings for each of the behaviors. The first rating was the self-reported frequency of performing the behavior (“How frequently do you do this activity”), which was considered as a proxy for habit strength. Responses were given on a 5-point scale ranging from “never” (1) to “always” (5). The second rating concerned the extent to which the behavior reflected participants’ true self. The instruction was to indicate “how much this activity is something that reflects *who you really are* as a person (your “true self”).” Responses were given on a 5-point scale ranging from “not at all” (1) to “very much” (5). For each individual participant, a correlation was calculated between the frequency and true self ratings across the 80 behaviors. These within-participant correlations were considered as a measure of individual differences in habit-identity associations.

##### Self-Esteem

Self-esteem was assessed by the 10-item Self-Esteem Scale (Rosenberg, 1965). Sample items are “I feel I have a number of good qualities” and “I wish I could have more respect for myself” (reverse-coded). Responses were given on 5-point scales ranging from “disagree” (1) to “agree” (5). Scores were coded such that higher numbers indicate higher self-esteem. Cronbach’s *α* was 0.85.

##### Self-Regulatory Focus

Individual differences in self-regulatory focus were assessed by the 18-item Promotion/Prevention Scale (Lockwood et al., 2002). The scale contains two subscales measuring a promotion and a prevention self-regulatory orientation, respectively. Examples of promotion orientation items are “I frequently imagine how I will achieve my hopes and aspirations” and “My major goal right now is to achieve my ambitions.” Examples of prevention orientation items are “I’m anxious that I will fall short of my responsibilities and obligations” and “My major goal right now is to avoid becoming a failure.” Responses were given on 7-point scales ranging from “not at all true of me” (1) to “very true of me” (7). Scores were coded such that higher numbers indicate a strong promotion or prevention focus. Cronbach’s *α*s were 0.87 and 0.73 for the promotion and prevention orientation subscales, respectively. The correlation between the two subscales was 0.42, *p* < 0.001. In order to investigate the unique variances of each subscale, uncorrelated factor scores for each subscale from a Varimax rotated factor analysis were used in the further analyses.

### Results and Discussion

The within-participant habit-identity correlations ranged from −0.19 to 0.89, suggesting substantial individual differences in habit-identity associations. The median correlation was 0.46. In the subsequent analyses, the habit-identity correlations were Fisher-*Z* transformed, although the results were nearly identical when untransformed correlations were used.

**Figure 1:**
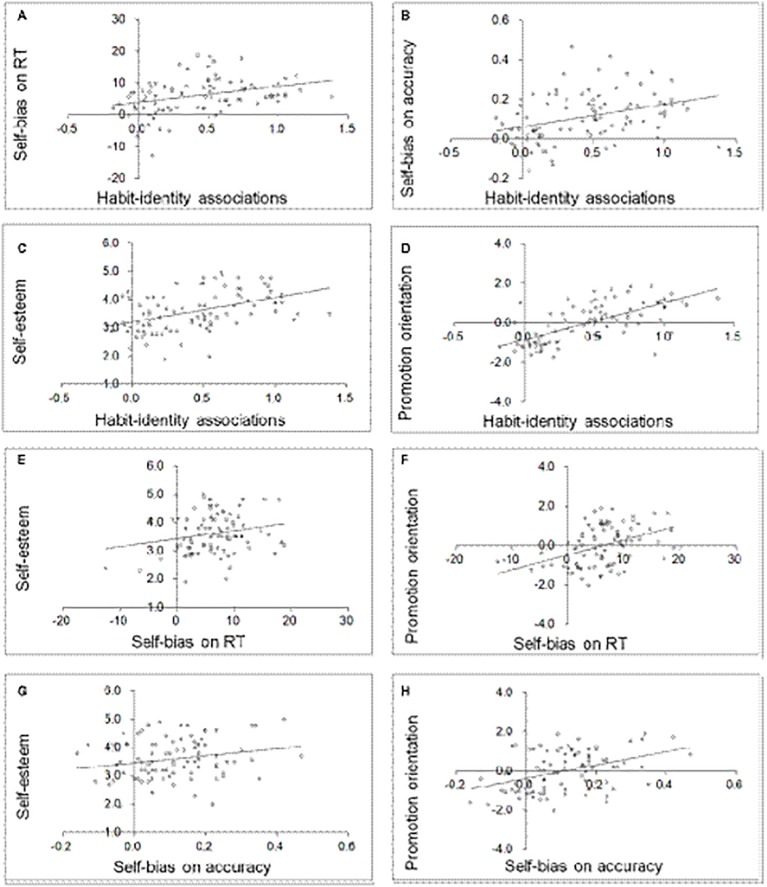
**(A–H)** Scatterplots of key correlations in Study 1.

In Table 1, means, standard deviations, and correlations between the study variables are presented. In Figure 1, the corresponding scatterplots of eight key correlations are shown. The results suggest that the degree to which individuals associated habits with self-identity correlated statistically significantly with both self-bias measures as well as with self-esteem and a promotion self-regulatory orientation. In addition, the self-bias measures correlated statistically significantly with self-esteem and a promotion orientation.

**Table 1:** Means, standard deviations, and correlations (Study 1).

Variable	*M*	SD	2	3	4	5	6
1. Habit-identity associations^1^	0.46	0.36	0.34***	0.34***	0.45***	0.68***	0.06
2. Self-bias on RT	6.23	5.34		0.51***	0.22*	0.40***	0.09
3. Self-bias on accuracy	0.11	0.12			0.21*	0.41***	0.05
4. Self-esteem	3.60	0.71				0.52***	−0.34***
5. Promotion orientation^2^	0.00	1.00					0.00
6. Prevention orientation^2^	0.00	1.00					

Note: N = 90. * = p < 0.05; *** = p < 0.001.

1 *Within-participant Fisher-Z transformed correlations*.

2 *Factor scores from a Varimax rotated solution. The means and standard deviations of the promotion and prevention raw scores were 5.13 (1.01) and 4.39 (0.88), respectively*.

Feelings of identity derived from habits were found associated with cognitive, affective, and motivational facets of the self. The pattern of correlations suggests that individuals for whom habits are strongly related to feelings of identity show stronger cognitive self-integration, higher self-esteem, and a stronger striving toward an ideal self. Note that the obtained correlations were between three very different types of data, that is, within-participant habit-identity correlations, latency/accuracy data, and self-assessments, respectively, which speaks against inflated correlations due to consistency and social desirability biases.

## Study 2

The assumption in Study 1 was that habits are implied in feelings of identity if these relate to important goals or values. Study 2 aimed to test that assumption. We contend that habit-identity associations are stronger if participants affirm the values that are perceived to be related to the respective habits. The habit-identity association task, which was used in Study 1, was thus presented under two conditions^1^. In a value affirmation condition, participants were asked for each of the 80 behaviors to indicate *why* they would do the activity, in addition to the habit and true self ratings. They could choose between 10 values, which represented the motivational continuum of Schwartz’s (1992) value circumplex. Participants in the control condition indicated for each activity at *which time* of the day they would likely engage in the activity and could choose between 10 specified times. The expectation was that the within-participant correlations between the habit and true self ratings would be stronger in the value affirmation versus control condition. The rationale was that value affirmation would enhance the salience of goals participants adhered to, which would thus lead to higher importance ratings.

### Method

#### Participants and Design

The study was conducted online *via* Prolific Academic, which is a UK-based platform for online studies. A power analysis was conducted prior to this study. As there are no previous studies that could serve as a benchmark, we aimed at being able to detect a small effect size in a two-sided *t* test between two independent samples (Cohen’s *d* ≈ 0.25), setting an *α* of 0.05, and accepting a power of 0.80. The sample size needed for this setup was approximately 500. A total of 500 participants were recruited, 482 of which completed the study. All participants were students. There were 307 males and 173 females, while two participants did not indicate a gender. Their mean age was 22 years (SD = 3.07). Informed consent was obtained from all participants according to procedures approved by the departmental ethics committee (IRB). Participants were randomly allocated to a value affirmation versus control condition. The task took 15–20 min to complete. Participants were paid £2.25 for their contribution.

#### Materials

The habit-identity association task contained the same 80 behaviors that were used in Study 1. As an explanation of habit ratings, participants were told: “How much of a habit is this activity for you? A habit is something you do frequently and automatically.” The ratings were then introduced as “When you have the opportunity, how frequently and automatically do you do this?^2^” Responses were given on a 5-point scale ranging from “never” (1) to “always” (5). As an explanation of true self ratings, participants were told: “How much does the activity reflect *who you really are* as a person? That is, to what extent does the activity represent what you would consider as your ‘true self.’” The identity ratings were then introduced as: “How much does this activity reflect your true self?” Responses were given on a 5-point scale ranging from “not at all” (1) to “very much” (5). In between each habit and identity rating, participants in the value affirmation condition were asked to choose from a pull-down menu *why* they would do the activity (“If you would do this, why?”). They were presented with 10 value areas (Schwartz, 1992), which were briefly explained: “Influence (social status and prestige, control over people and resources)”; “Achievement (personal success, competence, meeting high standards)”; “Pleasure (enjoyment, sensual gratification, indulgence)”; “Excitement (adventure, novelty, seeking challenges, exploring)”; “Independence (seeking freedom, independence, uniqueness, creativity)”; “Welfare (understanding, tolerance, welfare of people and nature)”; “Helpful (helping people you meet or are in frequent contact with)”; “Tradition (respect, commitment, acceptance of customs from culture or religion)”; “Conformity (abiding by the rules, meeting others’ expectations, respecting norms)”; “Security (safety, harmony, stability for yourself, others, and the community at large).^3^” The value labels and their descriptions were presented on an instruction page, while the pull-down menu contained the 10 value labels. In the control condition, participants were also presented with a pull-down menu but were asked to indicate *when* they would do the activity (“If you would do this, at what time would this typically occur?”). They could select one of the following 10 times: 7 AM, 9 AM, 11 AM, 1 PM, 3 PM, 5 PM, 7 PM, 9 PM, 11 PM, and 1 AM.

The validity of the value affirmation manipulation was tested in an online study among 93 participants. There were 38 males and 55 females, while two participants did not indicate a gender. Their mean age was 27 years (SD = 8.28). Informed consent was obtained from all participants according to procedures approved by the authors’ departmental ethics committee (IRB). Participants were presented with a random selection of 25 from the 80 behaviors and were randomly assigned to the value affirmation or control condition described above. For each behavior, they were asked how important this activity would be for them on a 6-point scale ranging from “not at all” (1) to “very much” (6). The 25 ratings were averaged. Participants in the value affirmation condition indeed gave higher importance ratings than participants in the control condition, *M*-value affirmation *=* 4.02, control *=* 3.73, *t*(91) = 2.25, *p* < 0.03, Cohen’s *d* = 0.47. This supported the validity of the value affirmation manipulation.

### Results and Discussion

The within-participant habit-identity correlations in this sample ranged from −0.21 to 0.99. The median correlation was 0.69. The median correlation was 0.71 in the value affirmation condition and 0.65 in the control condition. A *t* test was conducted after a Fisher-*Z* transformation of the correlations. The difference between the two conditions was statistically significant, *t*(480) = 2.34, *p* < 0.02, Cohen’s *d* = 0.21. The results were nearly identical when untransformed scores were used, *t*(480) = 2.58, *p* < 0.01, Cohen’s *d* = 0.23. This result provides proof of concept and suggests that habit-identity associations are stronger if habits are linked to value-based motivations.

## General Discussion

As we argued in the introduction, habits are not *necessarily* associated with identity. Individuals differ in which habits they develop, and thus in which habits, if any, make up part of their self-identity. Incidentally, we do not wish to argue that non-habitual behaviors cannot be part of someone’s self-identity. Our assumption was that some habits may be more prone to relate to feelings of identity than others, namely those habits that are instantiations of chronic goals or values. In the present studies, habits were selected that were inferred from basic value domains (Schwartz, 1992). As values are inherently motivational forces, those habits are more likely to be associated with value-related goals and have a higher likelihood to be central to the self and feelings of self-identity (e.g., Verplanken and Holland, 2002). The variation in the habit-identity association measure used in both studies demonstrated that there are individual differences in the degree to which people associate habits with self-identity. In Study 1, this variation correlated with cognitive self-integration (self-prioritization), self-esteem, and a promotion-style self-regulatory focus. Study 2 demonstrated that habit-identity associations are stronger when these are explicitly considered as instantiations of values, which corroborate the assumption that value-related habits are implied in feelings of self-identity.

The correlations found in Study 1 are consistent with integrated self frameworks as suggested by Kuhl et al. (2015) and Sui and Gu (2017), which stress the interactions between cognitive, affective, and motivational aspects of the self for control of behavior. The correlations with habit-identity associations suggest that perceiving oneself to do things that fulfill important goals may be part of such a network and may thus add to feelings of self-worth and represent strivings toward an “ideal self.” The latter may also be a source of positive emotional experiences, as positive emotions and higher self-esteem are consequences of successful promotion-oriented self-regulation (e.g., Higgins, 1998). Consistent with this, breaking down intrinsic associations between self and positivity leads to reduced performance in self-recognition (Ma and Han, 2010), and negative mood induces a decreased self-prioritization effect in perception (Sui et al., 2016). Kuhl et al. (2015) considered self-positivity and inner security as one of the functional characteristics of the integrated self. The positive relations found in Study 1 may thus point to what Rogers (1961) described as characterizing “a fully functioning person,” that is, someone who aims at fulfilling their full potential. While the individual components that were included in this study are interesting in their own right, the apparent relationships between these different pieces of data suggest such a more holistic integrative structure. Self-perception of habits and associated feelings of identity may thus play a role in this system, at least to the extent to which an integrated self has been developed. It should be noted though that a strong integrated self is not *necessarily* positive or wholesome but may also characterize individuals who are highly delusional or be associated with narcissism and self-aggrandizing. But in those individuals too, self-perception of habits may function to support such beliefs.

An important question is what exactly the underlying mechanisms are of an integrated self. In other words, what are the dynamics that govern the relationships between behavioral, cognitive, affective, and motivational facets of an integrated self? The correlational data of Study 1, while demonstrating relations between these entities, leave unanswered questions of causality. For instance, do stronger habit-identity associations contribute to stronger cognitive self-integration and positive self-feelings, or do individuals with a strong integrated self and high self-esteem become more attentive to what they are doing to fulfill their ideal self? A promising approach to model these relationships is provided by control-process models, which describe how individuals self-regulate in terms of behavior, cognition, affect, and motivation (e.g., Carver and Scheier, 1998; Vohs and Baumeister, 2017). While elaboration on these models is beyond the scope of this article, they describe processes that unfold when individuals experience discrepancies between a current state and a goal. Moral values that make up part of one’s true self may constitute such goals. If and when the self is activated, habits may fulfill different roles in a control-process model, for instance, as a way to lower the perceived discrepancy between a current state and a goal and thus generate positive affect. Habits may also function as a standard against which goal fulfillment is evaluated, which may lead to positive or negative feelings, depending on the outcome of such an operation. Another possible role of habits is a mechanism for the mind to prioritize the action from a range of options, which would lead to goal fulfillment (e.g., Verplanken et al., 1994).

In both studies, we correlated participants’ habit ratings with the degree to which they perceived these behaviors to be part of their true self. While the true self is experienced as highly personal and is fundamental to who a person thinks they are (e.g., De Freitas et al., 2018), the content of the moral beliefs, which underlie the true self, are strongly anchored in the culture the person belongs to (e.g., De Freitas et al., 2017). This makes the true self an inherently social construct. A specific habit (e.g., helping an elderly person) may thus constitute a course of action by which a culturally determined moral value (benevolence) is expressed. Habits that are strongly associated with moral values may thus function as benchmarks to evaluate not only oneself but also to make inferences, and indeed, judgments, about other people’s personality, mental state, or behavior (e.g., Newman et al., 2014, 2015; De Freitas et al., 2018).

A limitation of the present studies is that, for the obvious reason of avoiding an overload for participants, the habit and identity assessments for the 80 behaviors had to be confined to one-item measures, while for psychometric reasons, this is not ideal. A related, and arguably more fundamental, limitation is that the one-item measures of behavioral frequency leave room for the argument that we measured frequent, repetitive, or familiar behaviors, which may or may not be habitual according to the contemporary definitions of habit. This has been salvaged somewhat in Study 2 by assessing how “frequent and automatic” the behaviors were executed (but see Gardner and Tang, 2014). While we acknowledge this limitation, it has been demonstrated in numerous studies that used the Self-Report Habit Index (Verplanken and Orbell, 2003), which contains items assessing the experience of repetition as well as automaticity, that these two components are strongly correlated.

As a corollary, the present study contributes to a discussion with respect to the Self-Report Habit Index (SRHI; Verplanken and Orbell, 2003). One of the 12 items of this scale refers to self-identity (“Behavior X is something that is typically me”). It has been debated whether this item should be part of a self-assessment of habit (e.g., Gardner et al., 2012; Rebar et al., 2018). Apart from the fact that this item consistently shows high item-total correlations with the scale, the present findings support the validity of the item as part of the SRHI.

Insight into the relationship between habit and identity may have important implications for behavior change interventions, in particular the longevity of a change if an intervention is successful. Two conditions may have to be fulfilled for behavior change to be maintained over time. The first is to turn new behavior into a habit, that is, behavior that is executed frequently and automatically (e.g., Rothman et al., 2009; Walker et al., 2015; Gardner and Lally, 2018). But second, long-term behavior maintenance may be enhanced if a habit becomes part of an individual’s self-identity. For instance, West (2006) posits that self-identity can be a major driver of behavior change and, importantly, the maintenance of newly acquired behavior (e.g., Tombor et al., 2015). The present studies may thus point to an exciting new direction in designing more effective behavior change interventions, namely not only changing behavior per se but also turning new behavior into habits that are embedded in a self-identity context, and thus capitalize on an integrated self framework.

### Conclusion

Some habits serve a self-identifying purpose, in particular when these are considered in the context of self-related goals or central values. The self may function as a subjective center of gravity, involving cognitive, affective, motivational, and behavioral facets (e.g., Sui, 2016). The strength of this “gravitational force” differs between individuals. For some, the self seems a relatively loosely assembled structure, whereas for others, it has a much stronger coherence. The present studies suggest that for the latter type of individuals habits may play a role in this structure and thus make up part of one’s self-identity.

## Ethics Statement

This study was carried out in accordance with the recommendations of the British Psychological Society, with written informed consent from all subjects. All subjects gave written informed consent in accordance with the Declaration of Helsinki. The protocol was approved by the Departmental Ethics Committee, Department of Psychology, University of Bath (reference numbers #17-266 and #18-235 for Studies 1 and 2, respectively).

## Author Contributions

BV and JS contributed equally to the research and manuscript.

### Conflict of Interest Statement

The authors declare that the research was conducted in the absence of any commercial or financial relationships that could be construed as a potential conflict of interest.

## Footnotes

^1^None of the other assessments in Study 1 were included in this study.

^2^We used “frequently and automatically” in Study 2 instead of “frequently” in Study 1, because, on reflection, the former is more aligned with contemporary conceptions of habit (e.g., Verplanken and Orbell, 2003; Gardner, 2015).

^3^Some of the labels for the value areas were slightly adapted from the original labels Schwartz (1992) presented, as some of the latter were found too abstract for the purpose of this study (e.g., we used “helpful” instead of “benevolence”, and “influence” instead of “power”).
